# Detailed analysis of inner ear malformations in CHARGE syndrome patients – correlation with audiological results and proposal for computed tomography scans evaluation methodology

**DOI:** 10.1016/j.bjorl.2023.101383

**Published:** 2023-12-23

**Authors:** Agata Szleper, Magdalena Lachowska, Tomasz Wojciechowski, Katarzyna Pronicka-Iwanicka

**Affiliations:** a Department of Otorhinolaryngology, Head and Neck Surgery, Medical University of Warsaw, Poland; b Department of Otolaryngology, Audiology and Phoniatrics, Children's Memorial Health Institute, Warsaw, Poland; c Department of Descriptive and Clinical Anatomy, Medical University of Warsaw, Poland; d Department of Medical Genetics, Children's Memorial Health Institute, Warszawa

**Keywords:** Inner ear, Hearing loss, Internal auditory canal, Semicircular canal, Temporal bone

## Abstract

**Objectives:**

The aim was to describe the spectrum of inner ear malformations in CHARGE syndrome and propose a Computed Tomography (CT) detailed scan evaluation methodology. The secondary aim was to correlate the CT findings with hearing thresholds.

**Methods:**

Twenty ears of ten patients diagnosed with CHARGE syndrome were subjected to CT analysis focusing on the inner ear and internal acoustic canal. The protocol used is presented in detail. ASSR results were analyzed and correlated with inner ear malformations.

**Results:**

Cochlear hypoplasia type III was the most common malformation found in 12 ears (60%). Cochlear hypoplasia type II, aplasia with a dilated vestibule, and rudimentary otocyst were also identified. In 20%, no cochlear anomaly was found. The lateral Semicircular Canal (SCC) absence affected 100% of ears, the absence of the posterior SCC 95%, and the superior SCC 65%. Better development of cochlea structures and IAC correlated significantly with the lower hearing thresholds.

**Conclusion:**

This study demonstrated that rudimentary SCC or a complete absence of these SCCs was universally observed in all patients diagnosed with CHARGE syndrome. This finding supports the idea that inner ear anomalies are a hallmark feature of the CHARGE, contributing to its distinct clinical profile. The presence of inner ear malformations has substantial clinical implications. Audiological assessments are crucial for CHARGE syndrome, as hearing loss is common. Early detection of these malformations can guide appropriate interventions, such as hearing aids or cochlear implants, which may significantly improve developmental outcomes and communication for affected individuals. Recognizing inner ear malformations as a diagnostic criterion presents implications beyond clinical diagnosis. A better understanding of these malformations can advance the knowledge of CHARGE pathophysiology. It may also help guide future research into targeted therapies to mitigate the impact of inner ear anomalies on hearing and balance function.

**Level of evidence:**

4.

## Introduction

CHARGE syndrome is a non-random congenital anomalies association that was first independently described in the medical literature in 1979 by Hall and Hittner,^1^, ^2^ then updated by Blake et al. in 1998^3^ and Verloes in 2005.^4^ It is a rare syndrome estimated to occur in 1:10,000 to 1:17,000 births, depending on the region.^5^, ^6^ CHARGE is an acronym for Coloboma, Heart defects, Atresia of the choanae, Retardation of growth and development, Genital and urinary abnormalities, Ear abnormalities, and/or hearing loss. According to Blake et al.,^3^ more precise criteria for diagnosis can be distinguished. They have been divided into three groups: major criteria, minor criteria, and occasional findings. Ear anomalies are included in the major criteria.

According to Blake et al., more than 90% of children with CHARGE syndrome have hearing impairment. The most common defects in the middle ear include abnormalities of the ossicle chain, up to 80%, and aberrant course of the facial nerve, covering almost three-quarters of patients.^7^ Inner ear malformations are combinations of cochlea, semicircular canals, and vestibule defects and will be discussed in more detail in this study.

In consequence, the complexity of the previously mentioned defects, together with the frequent co-occurrence of cleft palate (15%–20%) and choanal atresia (50%–60%) in CHARGE patients, may predispose to the poor outcome of conservative treatment.^3^ Audiological testing of patients with CHARGE consists of objective tests, including Auditory Brainstem Responses (ABR) and, when available, Auditory Steady-State Responses (ASSR). Audiological diagnosis is carried out early regarding the child's age, allowing for objective audiological tests during physiological, unsedated sleep.

## Aim

Our study aimed to describe the spectrum of inner ear malformations in CHARGE syndrome patients and propose a Computed Tomography (CT) detailed scan evaluation methodology. The secondary aim was to correlate the computed tomography findings with hearing thresholds measured with Auditory Steady-State Responses (ASSR).

## Methods

### Ethical consideration

This retrospective study was approved by the local Institutional Ethics Committee (decision number AKBE/175/2022). The project conforms to The Code of Ethics of the World Medical Association (Declaration of Helsinki).

### General study group information

This study performed a retrospective analysis of data obtained from the medical records of patients diagnosed with CHARGE syndrome. Twenty temporal bones of ten consecutive patients diagnosed with the syndrome were subjected to detailed CT analysis focusing on the inner ear and internal acoustic canal. One patient did not have a complete set of audiological tests; however, he was included in the study group due to the CT findings worth detailed radiological assessment. Two independent, experienced investigators (also authors of this manuscript) evaluated the patient's CT scans using the same study protocol and questionnaire.

The patients were diagnosed in two large co-working tertiary centers due to a typical CHARGE combination of clinical features. One center provides genetic and audiological testing and is a full children's tertiary hospital (covering all specialties needed for CHARGE syndrome patients and more). The second one specializes in audiological testing and hearing prostheses, including cochlear implants in children. According to the literature, about 60% of patients with CHARGE present autosomal dominant mutations in the CHD7 gene. More than 1000 mutation variants that can cause CHARGE syndrome have been identified.^6^, ^8^, ^9^, ^10^, ^11^, ^12^ In this study, six analyzed patients underwent genetic testing confirming the gene mutations. Four were not analyzed genetically due to parents' no agreement with these tests but presented typical specific and recognizable patterns of CHARGE anomalies.

All ten patients underwent CT scanning of the temporal bone. This study included only the medical records of patients with CHARGE syndrome and CT scans. Patients with CHARGE syndrome diagnosis but no CT were not included. Performing full audiological diagnostics and CT scanning of the temporal bone, which in most cases in small children requires sedation, is not always a priority due to the other more serious components of the syndrome.

### Computed tomography scans analysis procedure in detail

A scan analysis protocol scheme was used for CT evaluation, which had proven satisfactory in previous studies. In addition, a questionnaire with key features was created to assist the investigators with CT evaluation and to standardize their assessment and thus enable the comparison of results. Table 1 presents the mentioned questionare, pointing out the key features of computed tomography analysis. An attempt was made to classify the identified malformations using the newest Sennaroglu classification.^13^

**Table 1:** Results of computed tomography of the inner ear detailed evaluation in patients with CHARGE syndrome.

Patient Nº	Ear	Vestibule	Lateral semicircular canal	Posterior semicircular canal	Superior semicircular canal	IAC diameter (mm)	Basal turn	Middle and apical turns	Modiolus	Cochlear malformation type
**1**	**R**	Normal	Absent	Absent	Absent	6.08	Normal	Shortened, less than 2 turns	Present	Cochlear hypoplasia type III
**1**	**L**	Normal	Absent	Absent	Absent	3.76	Normal	Shortened, less than 2 turns	Present	Cochlear hypoplasia type III
**2**	**R**	Hypoplastic	Absent	Absent	Hypoplastic	2.70	Normal	Cystic, hypoplastic	Absent	Cochlear hypoplasia type II
**2**	**L**	Hypoplastic	Absent	Absent	Absent	2.27	Normal	Cystic, hypoplastic	Absent	Cochlear hypoplasia type II
**3**	**R**	Hypoplastic	Absent	Absent	Hypoplastic	3.72	Normal	Shortened, less than 2 turns	Present	Cochlear hypoplasia type III
**3**	**L**	Hypoplastic	Absent	Absent	Hypoplastic	3.22	Normal	Shortened, less than 2 turns	Present	Cochlear hypoplasia type III
**4**	**R**	Normal	Absent	Absent	Absent	4.45	Normal	Shortened, less than 2 turns	Present	Cochlear hypoplasia type III
**4**	**L**	Normal	Absent	Absent	Absent	4.22	Normal	Shortened, less than 2 turns	Present	Cochlear hypoplasia type III
**5**	**R**	Normal	Absent	Absent	Absent	3.46	Normal	Shortened, less than 2 turns	Present	Cochlear hypoplasia type III
**5**	**L**	Normal	Absent	Absent	Absent	2.86	Normal	Shortened, less than 2 turns	Present	Cochlear hypoplasia type III
**6**	**R**	Hypoplastic	Absent	Absent	Hypoplastic	3.14	Normal	Normal	Present	Isolated vestibular labyrinth anomaly
**6**	**L**	Hypoplastic	Absent	Absent	Absent	3.85	Normal	Normal	Present	Isolated vestibular labyrinth anomaly
**7**	**R**	Hypoplastic	Absent	Absent	Absent	3.59	Normal	Shortened, less than 2 turns	Present	Cochlear hypoplasia type III
**7**	**L**	Hypoplastic	Absent	Absent	Absent	3.18	Normal	Shortened, less than 2 turns	Present	Cochlear hypoplasia type III
**8**	**R**	Hypoplastic	Absent	Absent	Absent	3.22	Normal	Normal	Present	Isolated vestibular labyrinth anomaly
**8**	**L**	Hypoplastic	Absent	Absent	Absent	3.36	Normal	Shortened, less than 2 turns	Present	Cochlear hypoplasia type III
**9**	**R**	Hypoplastic	Absent	Absent	Hypoplastic	4.51	Normal	Normal	Present	Isolated vestibular labyrinth anomaly
**9**	**L**	Hypoplastic	Absent	Absent	Hypoplastic	3.77	Normal	Shortened, less than 2 turns	Present	Cochlear hypoplasia type III
**10**	**R**	Dilated	Absent	Absent	Dysmorphic	0	Aplasia	Aplasia	Aplasia	Cochlear aplasia with a dilated vestibule (CADV)
**10**	**L**	Rudimentary otocyst	Absent	Absent	Absent	0	Rudimentary otocyst	Rudimentary otocyst	Rudimentary otocyst	Rudimentary otocyst

IAC, Internal Acoustic Canal.

All scans were analyzed using RadiAnt DICOM Viewer 2022.1 64-bit (Medixant, Poznan, Poland) with the MultiPlanar Reconstruction (MPR) option.

Initially, in each case, the researcher set the window level to 500 Hounsfield Units (HU) and the window width to 3000 HU. Then, the protocol developed in earlier studies was used as follows. The MPR tool assessed the semicircular canals in three reference planes. Initially, the axial plane in which the Lateral Semicircular Canal (LSC) should show a typical “signet ring appearance”. Then, investigators searched for the plane of the Anterior Semicircular Canal (ASC, as described by Pöschl) and the Posterior Semicircular Canal (PSC) with common crus.

The next challenge was to identify and describe the cochlea and vestibule. At first, the longitudinal section of the cochlea was set to assess the basal, middle, and apical turns around the modiolus. Afterward, the adjusted axial plane was set at an angle that allowed for the evaluation of the modiolus and basal turn of the cochlea. Subsequently, a perpendicular plane was defined concerning the modified axial plane, enabling the visualization of the modiolus and all three cochlea turns. However, due to the severity of malformations in analyzed patients, the reference points for determining such planes were disturbed, making the analysis much more difficult.

Internal Acoustic Canal (IAC) diameter was assessed in an axial section of CT by performing measurements at the level of the internal acoustic pore. Firstly, a longitudinal axis line was drawn, and then the IAC diameter was measured as a line drawn perpendicularly to the longitudinal axis of the IAC.

**Figure 1:**
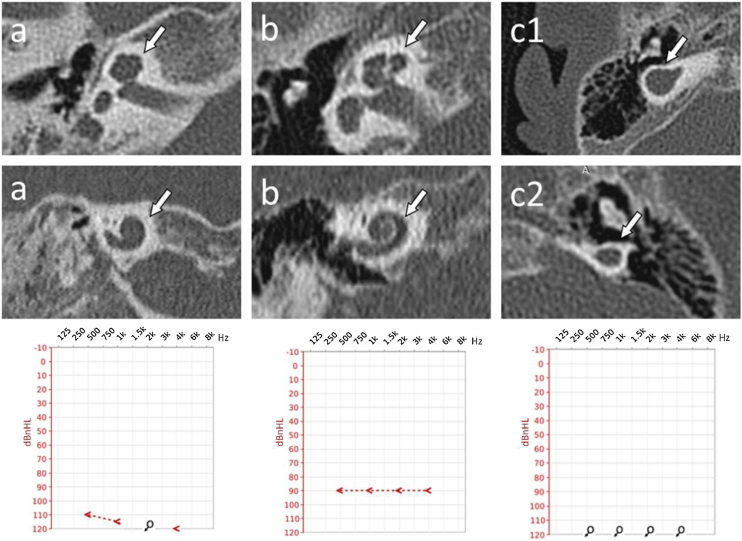
Cochlear malformations identified in computed tomography evaluation and presented in the planes selected for the most precise visualization. Each panel represents a particular patient: (a) Case #2 right ear, cochlear hypoplasia type II; (b) Case #1 right ear, cochlear hypoplasia type III; (c) Case #10 right ear, (c1) Cochlear Aplasia with a Dilated Vestibule (CADV); (c2) Rudimentary otocyst. White arrows indicate anomaly location. Individual cases are supplemented with ASSR reconstruction of audiograms (hearing thresholds in dBnHL), respectively.

**Figure 2:**
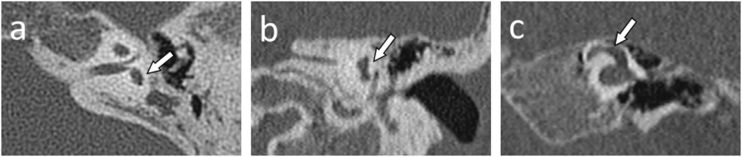
Semicircular canals and vestibule malformations identified in computed tomography evaluation and presented in dedicated planes. Panel a and b) represent the same patient (case #2) with a hypoplastic vestibule and the absence of all three semicircular canals. Panel c (case #10) shows a dysmorphic superior semicircular canal with an accompanying dilated vestibule.

**Figure 3:**
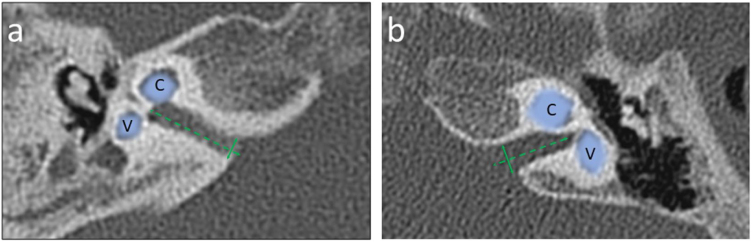
Exemplary measurements of the Internal Auditory Canal (IAC) diameter in an axial section of a computed tomography made at the internal acoustic opening (*porus acusticus internus)*, the IAC diameter (solid line) was determined perpendicular to the longitudinal axis (dashed line) of the IAC; Panel a – narrowed IAC with diameter 2.70 mm (case #2); Panel b ‒ normal sized IAC with diameter 3.76 mm (case #1). Cochlea (C) and Vestibule (V) are marked with a line art over for easier identification.

For better visualization and understanding of found malformations in the inner ear in analyzed patients with CHARGE syndrome, examples are shown in Figure 1, Figure 2, Figure 3.

### Audiological assessment

The assessment of the patient's hearing was carried out in a way suitable for their developmental stage and age. Audiological test results of hearing thresholds (ASSR) were available for analysis in 9 patients (18 ears) and were performed at the age between 3 months to 2.5 years. The results confirmed hearing loss. ASSR results for 500, 1000, 2000, and 4000 Hz were analyzed for the purposes of this study. In ASSR, if the threshold for a particular frequency was absent for the highest intensity level tested (120 dBnHL result as no response), its value was set as 130 dBnHL to facilitate the analyses.

### Statistical analysis

Statistical analysis was conducted in the STATISTICA program (TIBCO Software Inc. 2017, version 13.3). The data were tested for normality, parametric, and non-parametric criteria. Detailed statistical analysis was performed using Spearman's correlation test. The level of statistical significance was set at *p* = 0.05.

## Results

Twenty temporal bones were analyzed. Two males and eight females were among the investigated children with CHARGE syndrome. As mentioned earlier, one patient did not have a complete set of audiological tests in his medical records (ASSR results were missing). However, he was included in the study group due to the findings worth detailed assessment in CT.

### Computed tomography findings

The structure of the cochlea was considered in terms of the basal turn and then jointly in terms of the middle and apical turns. Moreover, the absence or presence of modiolus was assessed. Then, the findings were summarily classified to a specific malformation type according to the newest Senneroglu classification.^13^ The basal turn was diagnosed as normal in 90% of cases. However, one patient with cochlear aplasia and rudimentary otocysts was also identified. More variety in structure was found in the middle and apical turns. Besides cochlear aplasia and rudimentary otocyst, the following abnormalities were discovered, which are essential concerning the entire bony labyrinth. Respectively: 2 cases (10%) of dysmorphic (cystic) middle and apical turns, 12 cases (60%) of shortened (less than two turns), and 4 cases (20%) of normal, fully developed cochlea. Modiolus was absent in 20% of cases. In the rest of the cases, the modiolus was considered present. Cochlear anomalies identified in analyzed patients are presented in Table 1, and examples are shown in Fig. 1.

Lateral semicircular canal defects were the most common anomaly in analyzed patients with CHARGE syndrome, affecting 100% of cases. The second most common anomaly concerned the absence of the posterior semicircular canal in all temporal bones except one, where the canal was preserved in its dysmorphic form (5%). The superior semicircular canal was affected by a deep anomaly to a slightly lesser extent. Its absence was observed in 13 temporal bones (65%). In 6, it was hypoplastic (30%), and in 1 case – dysmorphic (5%). However, the vestibule itself was normal in 6 cases (30%), hypoplastic in 12 cases (60%) and absent in one case (5%). Also, the vestibule was dilated in one case, which coexisted with cochlear aplasia. Semicircular canals defects and vestibular anomalies identified in analyzed patients are presented in Table 1, and examples are shown in Fig. 2.

Cochlear Aplasia with a Dilated Vestibule (CADV) and rudimentary otocyst was accompanied by the absence of IAC, which for statistical analysis purposes in this study was considered 0.00 mm in diameter. In the remaining cases, the IAC diameter ranged from a minimum dimension of 2.27 mm–6.08 mm. The median IAC diameter of all ears was 3.41 mm, and the Standard Deviation (SD) was 1.37 mm. IAC measurement examples are shown in Fig. 3.

### Classification of inner ear malformations

In the final stage of the CT investigation, the analysis of individual elements with a summary aimed at assigning specific malformations to each case according to the Senneroglu classification^13^ was carried out. Cochlear Aplasia with a Dilated Vestibule (CADV) and rudimentary otocyst (Fig. 1c) was seen in both ears in one patient (10% of the analyzed ears, patient #10). It was also related to the almost complete aplasia of the semicircular canals. Only in the right ear was a structure that could be regarded as a remnant of the dysplastic superior semicircular canal (Fig. 2c). Cochlear hypoplasia type II (Fig. 1a) was also seen in both ears in one patient (10% of the analyzed ears, patient #2). Cochlear hypoplasia type III (Fig. 1b) was the most common malformation found in 12 ears (60%). In 4 cases, no cochlear anomaly was identified. Still, in half of them, normal cochlea was accompanied by isolated semicircular canals anomaly and, in two other cases, by a hypoplastic vestibule. In two patients, the defects were not symmetrical in both ears. In both of them, the right ear was more severely affected with noticeable cochlear hypoplasia type III, while the left ear showed only isolated vestibular labyrinth anomaly.

In addition, some correlations were observed between the severity of malformations of individual structures. Firstly, the correlation between the IAC diameter and the severity of vestibular malformations was statistically significant. The more severe the vestibule malformation, the smaller the IAC diameter (*p* = 0.013). In addition, the severity of vestibular defect significantly correlated with the severity of hypoplasia of the basal turn of the cochlea (*p* = 0.0057). Moreover, the correlation occurred in the combination of vestibular malformation with the presence or absence of modiolus. Its absence correlated with the vestibular malformation (*p* = 0.0125).

### Audiological findings

Audiological results were unavailable for one patient (patient #6). The remaining nine patients underwent audiological evaluation. The results are shown in Table 2. The ASSR results have been converted to reconstructed audiograms for better understanding and visualization. In the ASSR, there was a statistically significant correlation between the severity of the inner ear malformation and the profoundness of the hearing loss for all of the frequencies, with *p-*values ranging from 0.0001 to 0.0432. The most advanced malformations, such as Cochlear Aplasia with a Dilated Vestibule (CADV) and rudimentary otocyst, showed non-response, which is understandable considering not only the condition of the cochlea (absence) but the absence of IAC on both sides as well. Subsequently, type II hypoplasia correlated with worse hearing level outcomes than type III hypoplasia. Ultimately, patients with the best-developed cochlea had the best audiological results. When inspecting the structure of the cochlea more deeply, taking into consideration the state of the basal, middle, and apical turns and the presence or absence of the modiolus, it was also observed that the better development of these structures, the hearing thresholds become lower (*p-*value ranged from 0.0000 to 0.0432). A significant correlation was also observed for IAC morphology, where absence, narrowing, or normal size correlated statistically significantly with audiological results (*p-*value ranged from 0.0001 to 0.0002). Moreover, a significant correlation with the ASSR scores was observed regarding the exact dimension of the IAC (*p-*value ranged from 0.0076 to 0.0288). The results of the statistical analysis are presented in Table 3.

**Table 2:** Auditory Steady-State Response (ASSR) results recorded from analyzed ears – hearing thresholds for frequencies 500, 1000, 2000, and 4000 Hz.

Patient Nº	Cochlear malformation type	Ear	ASSR 500 Hz (dBnHL)	ASSR 1000 Hz (dBnHL)	ASRR 2000 Hz (dBnHL)	ASRR 4000 Hz (dBnHL)
1	Cochlear hypoplasia type III	R	90	90	90	90
1	Cochlear hypoplasia type III	L	100	100	100	100
2	Cochlear hypoplasia type II	R	110	115	NR	120
2	Cochlear hypoplasia type II	L	115	125	NR	NR
3	Cochlear hypoplasia type III	R	65	70	75	75
3	Cochlear hypoplasia type III	L	65	80	75	70
4	Cochlear hypoplasia type III	R	85	100	90	90
4	Cochlear hypoplasia type III	L	35	40	25	15
5	Cochlear hypoplasia type III	R	80	70	60	60
5	Cochlear hypoplasia type III	L	100	100	100	100
6	Isolated vestibular labyrinth anomaly	R	NA	NA	NA	NA
6	Isolated vestibular labyrinth anomaly	L	NA	NA	NA	NA
7	Cochlear hypoplasia type III	R	75	95	70	80
7	Cochlear hypoplasia type III	L	85	90	80	70
8	Isolated vestibular labyrinth anomaly	R	35	50	45	45
8	Cochlear hypoplasia type III	L	75	90	90	90
9	Isolated vestibular labyrinth anomaly	R	45	65	70	70
9	Cochlear hypoplasia type III	L	60	65	70	70
10	Cochlear Aplasia with a Dilated Vestibule (CADV)	R	NR	NR	NR	NR
10	Rudimentary otocyst	L	NR	NR	NR	NR

NR, No Response up to 120 dBnHL; NA, Not Applicable.

**Table 3:** Spearman rank correlation results between inner ear anomaly severity and hearing thresholds determined in Auditory Steady-State potentials (ASSR) for 500, 1000, 2000, and 4000 Hz.

Analyzed pair of variables	Number of ears	R Spearman	*p-*value
IAC (Absent-1/Narrow-2/Normal-3) & ASSR 500 Hz	18	−0.7788	0.0001*
IAC (Absent-1/Narrow-2/Normal-3) & ASSR 1000 Hz	18	−0.7703	0.0002*
IAC (Absent-1/Narrow-2/Normal-3) & ASRR 2000 Hz	18	−0.7713	0.0002*
IAC (Absent-1/Narrow-2/Normal-3) & ASRR 4000 Hz	18	−0.7775	0.0001*
IAC diameter (mm) & ASSR 500 Hz	18	−0.5927	0.0095*
IAC diameter (mm) & ASSR 1000 Hz	18	−0.6068	0.0076*
IAC diameter (mm) & ASRR 2000 Hz	18	−0.5705	0.0134*
IAC diameter (mm) & ASRR 4000 Hz	18	−0.5149	0.0288*
Basal turn (Aplasia-1/R-otocyst -2/Normal-3) & ASSR 500 Hz	18	−0.5459	0.0191*
Basal turn (Aplasia-1/R-otocyst -2/Normal-3) & ASSR 1000 Hz	18	−0.5473	0.0187*
Basal turn (Aplasia-1/R-otocyst -2/Normal-3) & ASRR 2000 Hz	18	−0.4812	0.0432*
Basal turn (Aplasia-1/R-otocyst -2/Normal-3) & ASRR 4000 Hz	18	−0.5153	0.0286*
Middle and apical turns (Aplasia-1/R-otocyst-2/Dysmo-3/Shortened-4/Normal-5) & ASSR 500 Hz	18	−0.8138	0.0000*
Middle and apical turns (Aplasia-1/R-otocyst-2/Dysmo-3/Shortened-4/Normal-5) & ASSR 1000 Hz	18	−0.7986	0.0001*
Middle and apical turns (Aplasia-1/R-otocyst-2/Dysmo-3/Shortened-4/Normal-5) & ASRR 2000 Hz	18	−0.7663	0.0002*
Middle and apical turns (Aplasia-1/R-otocyst-2/Dysmo-3/Shortened-4/Normal-5) & ASRR 4000 Hz	18	−0.7621	0.0002*
Modiolus (Absent-1/Present-2) & ASSR 500 Hz	18	−0.7234	0.0007*
Modiolus (Absent-1/Present-2) & ASSR 1000 Hz	18	−0.7253	0.0007*
Modiolus (Absent-1/Present-2) & ASRR 2000 Hz	18	−0.7288	0.0006*
Modiolus (Absent-1/Present-2) & ASRR 4000 Hz	18	−0.7284	0.0006*
Cochlear malformation type (Aplasia-1/R-otocyst2/Hypopl-II-3/Hypopl-III-4/Normal-5) & ASSR 500 Hz	18	−0.8138	0.0000*
Cochlear malformation type (Aplasia-1/R-otocyst2/Hypopl-II-3/Hypopl-III-4/Normal-5) & ASSR 1000 Hz	18	−0.7986	0.0001*
Cochlear malformation type (Aplasia-1/R-otocyst2/Hypopl-II-3/Hypopl-III-4/Normal-5) & ASRR 2000 Hz	18	−0.7663	0.0002*
Cochlear malformation type (Aplasia-1/R-otocyst2/Hypopl-II-3/Hypopl-III-4/Normal-5) & ASRR 4000 Hz	18	−0.7621	0.0002*

The “*p*” value represents the level of significance. The asterisks (*) and bold font were used to mark statistically significant correlations (*p* < 0.05).

R-otocyst, Rudimentary Otocyst; Dysmo, Dysmorphic; Hypopl-II, Hypoplasia type II; Hypopl-III, Hypoplasia type III.

## Discussion

Inner ear malformations are a common finding in individuals diagnosed with CHARGE syndrome. The present study highlights the prevalence and importance of these malformations, shedding light on their diagnostic significance and potential implications for clinical management.

Interestingly, the distribution of malformation types identified in different studies amongst CHARGE syndrome patients is not always convergent. In our study, the dominant malformation is cochlear hypoplasia type III. Less common are cochlear hypoplasia type II, cochlear aplasia, rudimentary otocysts, and isolated defects of the vestibular system. Vesseur et al.^14^ analyzed CHARGE syndrome patients and showed more than 60% of cases involved a normal cochlea with isolated semicircular canal defects. In addition, the second significant group was malformations that the researchers described as cochlear hypoplasia type IV, although not without discussion, partly due to a mismatch with any malformations considered. In our study, similar difficulties in identifying the cochlear defect occurred in case #2. The cochlea was initially classified as incomplete partition type II due to the confluence of the middle and apical turns. Later, with deeper CT evaluation using the proposed protocol in this study, the classification was changed to cochlear hypoplasia type II due to the lack of modiolus and poorly developed basal turn. A similar problem was encountered by Lewis et al., who decided to omit the classic division and classify cochlear hypoplasia into four subgroups: normal, mild, moderate, and severe, with the mild cochlear hypoplasia phenotype as the most common.^11^

In their study group, Aragón-Ramos et al.^15^ found cochlear hypoplasia type III as the most popular defect, consistent with our results. Da Costa Monsanto et al.^16^ found cochlear hypoplasia in all examined temporal bones from donors with CHARGE syndrome. Before Sennaroglu systematized inner ear malformations in 2017, researchers did not always use a unified description when analyzing defects in CHARGE patients. Rah et al.^17^ identified inner ear malformations in 82% of CHARGE patients undergoing cochlear implantation, collectively termed cochlear hypoplasia, which was consistent with similar work of Birman et al.,^18^ where broadly interpreted cochlear hypoplasia has been reported in 80% of implanted patients. However, significantly different results are presented by Vincenti et al.^19^ In their study, not one of the eight analyzed patients with CHARGE was found to have cochlear hypoplasia. The anomalies included incomplete partition types I and II, common cavity, and, in half of the cases, an isolated defect of the vestibular labyrinth. As their work focused on aspects of cochlear implantation, they did not present the methodology of CT evaluation used for assessing the inner ear structures.

The genetic background also seems to be not without significance in the following considerations. According to Zentner et al.,^9^ inner ear anomalies identified through temporal bone CT or skull X-Ray were significantly higher in individuals with CHD7 mutations (98%) than those without mutations (75%). Adding a genetic factor to the description of malformations could shed light on the observed discrepancies.

In all patients in our study group, the absence of semicircular canals was found to some extent. It aligns with other authors who even argued that it is a reliable and crucial factor for diagnostic purposes. According to Lalani et al.,^20^ a combination of coloboma, choanal atresia, and abnormal semicircular canals highly predicted the presence of a CHD7 mutation. Wineland et al.^21^ found semicircular canal hypoplasia/aplasia more prevalent than other characteristic features such as coloboma or choanal atresia in their study population. Sanlaville et al.^22^ showed that the absence of a semicircular canal is detectable by ultrasound and should be searched for in fetuses with conotruncal heart defects. Moreover, in 10 fetuses with CHD7 mutations, arhinencephaly and semicircular canal agenesis were two constant features. Kimura and Kaga^23^ found that children with CHARGE and semicircular canal aplasia presented severe dysfunction of the vestibular ocular reflex, which pointed to impaired functioning of the vestibular organ, which in turn may be associated with impaired motor development.

According to other studies in the literature, the width of the IAC correlates with the occurrence of inner ear malformations and bilateral sensorineural hearing loss.^24^, ^25^ Another study^16^ revealed that the width of the IAC in individuals with inner ear malformations did not show a significant correlation with the number of Spiral Ganglion Neurons (SGNs) or the presence of aplastic/hypoplastic cochlear nerves. Our study considered a narrowed IAC less than 3 mm wide. However, in some studies in the literature,^24^, ^25^, ^26^ sometimes the IAC width of 2 mm is considered the lower limitation of normal size.

All of the above mentioned studies from the literature, except for one, were based on small groups of patients due to the low prevalence of the discussed syndrome. The number of patients ranged from 6 to 13 cases. Only the Vesseur et al. study^14^ included a larger group of 42 patients. Still, the data analysis also included analog CT (data dating back to 1996), in which multiplanar reconstruction was impossible. The sample size was also a limitation of our study. A series of 20 ears could influence the generalizability of the findings, making it necessary to conduct larger, preferably multi-center studies to validate the prevalence of inner ear malformations in a broader population of individuals diagnosed with CHARGE syndrome. Further investigations exploring the specific molecular mechanisms underlying these malformations could provide valuable insights into the developmental processes disrupted in CHARGE syndrome.

## Conclusions

The results of this study demonstrate that rudimentary semicircular canals or a complete absence of these canals were universally observed in all patients diagnosed with CHARGE syndrome. This finding supports the idea that inner ear anomalies are a hallmark feature of the CHARGE syndrome, contributing to its distinct clinical profile.

The presence of inner ear malformations has substantial clinical implications. Audiological assessments are crucial for individuals with CHARGE syndrome, as hearing loss is a common feature associated with inner ear anomalies. Early detection of these malformations can guide appropriate interventions, such as hearing aids or cochlear implants, which may significantly improve developmental outcomes and communication for affected individuals. Recognizing inner ear malformations as a diagnostic criterion presents implications beyond clinical diagnosis. A better understanding of these malformations can advance the knowledge of CHARGE syndrome pathophysiology. It may also help guide future research into targeted therapies to mitigate the impact of inner ear anomalies on hearing and balance function. This study adds to the knowledge and diagnostics of patients with CHARGE syndrome for healthcare use.

## Declaration of Generative AI and AI-assisted technologies in the writing process

While preparing this work, the authors did not use AI or AI-assisted technologies.

## Conflicts of interest

The authors declare no conflict of interest, including any financial interest or support related to this manuscript. This research did not receive any specific grant from the public, commercial, or not-for-profit funding agencies.
